# Adaptation and Validation of the “Support and Control in Birth” (SCIB) Tool in Postpartum Spanish Women

**DOI:** 10.3390/jcm15072495

**Published:** 2026-03-24

**Authors:** Sergio Martínez-Vázquez, Rocío Adriana Peinado-Molina, Leticia Molina-García, Antonio Hernández-Martínez, Juan Miguel Martínez-Galiano

**Affiliations:** 1 Department of Nursing, University of Jaen, 23071 Jaen, Spain; svazquez@ujaen.es (S.M.-V.); letitedi@gmail.com (L.M.-G.); jgaliano@ujaen.es (J.M.M.-G.); 2 Consortium for Biomedical Research in the Epidemiology and Public Health (CIBERESP), 28029 Madrid, Spain; 3 University Hospital of Jaén, 23007 Jaen, Spain; 4 Department of Nursing, Physiotherapy and Occupational Therapy, Faculty of Nursing, University of Castilla-La Mancha, 13071 Ciudad Real, Spain; antonio.hmartinez@uclm.es

**Keywords:** birth experience, maternal support, perceived control, psychometric properties, scale validation, SCIB

## Abstract

**Background:** Maternal control and the sense of support significantly influence a woman’s experience of birth. This study aimed to adapt and validate the Support and Control in Birth (SCIB) scale in Spanish women to assess maternal perceptions of support and control during birth, and to develop and validate an abbreviated version of the instrument. **Methods**: A cross-sectional study was conducted with a sample of 302 Spanish women who had given birth within the previous 6 months and were at least 1 week postpartum. Content, construct, and criterion validity, as well as reliability, were analysed using an expert panel, Exploratory Factor Analysis (EFA), Confirmatory Factor Analysis (CFA), Cronbach’s Alpha Coefficient, and Intraclass Correlation Coefficient (ICC). Criterion validity was assessed using the Generalised Anxiety Disorder Screener (GAD-7) and the Birth Satisfaction Scale–Revised (BSS-R). **Results**: The KMO test yielded a value of 0.925, and Bartlett’s test of sphericity was significant (*p* < 0.001). EFA identified three factors (Support, External control, and Internal control) that explained 56.49% of the total variance. CFA showed good model fit for most of the evaluated indices. The SCIB scale correlated negatively with the GAD-7 and positively with the BSS-R (*p* < 0.001), as well as with several obstetric and neonatal variables (*p* < 0.05): planned pregnancy, high-risk pregnancy, onset and type of delivery, birth plan, use of epidural analgesia, maternal involvement, postpartum complications, and newborn characteristics. Cronbach’s alpha was 0.951, and the ICC indicated excellent consistency and agreement (0.995; 95% CI: 0.990–0.998). Based on expert panel consensus, a 24-item abbreviated version was developed that exhibited psychometric properties similar to those of the original version and a high correlation with it (r > 0.90). **Conclusions**: The Support and Control in Birth (SCIB) scale is a valid and reliable instrument for assessing perceptions of support and control during birth in Spanish women. The 24-item abbreviated version is recommended.

## 1. Introduction

The feelings of maternal control and perceived support are key predictors of how a woman experiences birth [1,2]. This event represents one of the most important milestones in a woman’s life [3]. Globally, one-third of women experience negative birth experiences [4,5].

Perceived control and support are influenced by various medical, obstetric, psychological, and interpersonal factors [6]. Negative experiences are related to clinical events, such as obstetric emergencies, episiotomies, or foetal complications [7]. From a psychological perspective, the sense of control and self-efficacy during labour are fundamental; maternal satisfaction depends largely on the ability to make decisions [2], manage pain independently [8], and maintain control in the birthing environment [9]. Negative birth perceptions serve as indirect markers of psychopathological risk, potentially triggering adverse psychological outcomes. These dimensions, related to the internal locus of control, are essential for satisfaction and directly impact maternal self-esteem, as well as the construction of a positive self-concept [10,11]. In the interpersonal sphere, the attention and support received from the partner and healthcare staff [12,13], effective communication with healthcare professionals [14], the use of non-pharmacological techniques to relieve pain during birth [8] and support at the start of breastfeeding [15] also play an important role in the overall perception of the birth experience.

The birth experience significantly influences the mother’s physical and emotional health [16], as well as the well-being of the newborn [17]. This experience remains in women’s memories for years [18]. A positive birth experience contributes to strengthening maternal self-esteem, generates a sense of accomplishment, and facilitates adaptation and development of the maternal role [19]. Furthermore, it is associated with a lower level of pain during birth and a shorter duration of labour [11]. In contrast, negative experiences can produce short- and long-term psychological consequences [17,20], manifesting in symptoms of post-traumatic stress disorder [16], depression [21], anxiety [22] and difficulties in the mother-child bond [23].

Although various tools exist to assess aspects related to the birth experience, such as expectations [24], quality of care [25], fear [24], stress [25] and participation during the process [26], the Support and Control in Birth (SCIB) scale, developed by Ford et al. [27], incorporates a unique multidimensional construct that comprehensively addresses, in a single scale, several aspects that influence the birth experience. This scale has been validated in other cultural contexts [11,28,29,30]. That addresses internal locus of control, external locus of control, and professional support in a single instrument: through dimensions: internal control, external control and support. While the SCIB primarily measures the birth experience across these dimensions, it can also be used as an indicator of psychopathological risk, since a negatively perceived birth can trigger adverse psychological outcomes. Currently, in the Spanish context, there is no validated tool that specifically measures these dimensions. Therefore, this study aims to validate the Spanish version of the SCIB scale. A significant novelty of this research is the development of an abbreviated 24-item version, providing a more efficient tool for clinical practice and future research to identify women at risk and develop evidence-based strategies and policies that promote a birthing process respecting maternal rights and expectations.

## 2. Methodology

A cross-cultural adaptation and validation study of the “Support and Control in Birth (SCIB)” scale into Spanish, following the guidelines developed by Ramada-Rodilla et al. [31], was carried out in 4 phases with a sample of postpartum women in Spain who gave birth in Spain. The Provincial Research Ethics Committee of Jaen approved the study (SICEIA-2024-000229). The women received a participant information sheet stating that participation was completely voluntary and that anonymity would be guaranteed. Those who agreed to participate signed the informed consent.

Phase 1: Translation and back-translation of the questionnaire

First, two bilingual translators, both native Spanish speakers, one of whom specialises in obstetrics, independently translated the questionnaire from English to Spanish. They were asked to rate the difficulty of translating each item on a scale of 0 to 10, with 0 being the easiest and 10 the hardest. No item received a score higher than 5. Both translations were then combined into a single version, which was given to two other bilingual translators, this time native English speakers, to independently back-translate the questionnaire into English [31].

Phase 2: Expert Panel

After obtaining the translated version, it was evaluated by a multidisciplinary panel of experts. Sixteen experts participated: six mothers and ten healthcare providers working in birth care (midwives and obstetricians). This group of experts came from various geographical areas of Spain in order to represent the diverse social, cultural, and linguistic realities of the country. They were contacted by email and invited to participate in the evaluation of the adaptation. After agreeing to participate, the Spanish adaptation was sent to them, and they were asked to evaluate each questionnaire item, assigning a score from 1 (worst) to 5 (best) for four parameters: wording, comprehension, relevance, and overall assessment. A section was also included for them to note any pertinent observations about each item. To reach consensus, the panellists’ median score had to be equal to 4 or higher. Once the evaluations were received, they were compiled, and the necessary corrections were made based on the experts’ feedback, resulting in version 1 of the questionnaire. The modified questionnaire was then sent back to the expert panel for a second evaluation, after which it received approval from all participants (version 2 of 33 items).

Phase 3: Piloting the questionnaire

The questionnaire was administered to 30 women who had given birth within the previous three months. In addition to administering the SCIB instrument, all the necessary questions for the validation process were also applied to this group of women, with the aim of improving its wording and clarity. Following the pilot study, two items were modified, resulting in version 2 of the questionnaire.

Phase 4. Application of the instrument to the target population to determine its psychometric properties

### 2.1. Design and Selection

A cross-sectional study was conducted in 2025 with Spanish women who met the following inclusion criteria: women who had given birth within the previous six months and were at least 1 week postpartum. Women under 18 years of age and those who did not understand Spanish were excluded. The sample size was estimated according to the criteria for conducting a factor analysis. These criteria consider between 4 and 10 subjects per item [32]; given that the SCIB scale contains 33 items, a minimum sample size of 132 women was required.

### 2.2. Information Sources

Data were collected using a questionnaire that included sociodemographic variables, obstetric history, details of the last delivery, obstetric practices performed, and neonatal outcomes. It also included anxiety-related measurements using the Generalised Anxiety Disorder Screener (GAD-7) [33,34] and satisfaction with birth (Birth Satisfaction Scale–Revised) (BSS-R) [35].

The scale being validated is the Support and Control in Birth scale (SCIB), which has demonstrated good psychometric properties (α = 0.95), both in its original study [27] and in previous research carried out by other authors [11,28,29,30]. The SCIB scale consists of 33 items, divided into three subscales that assess internal control (items 1–10, including “I was in control of my emotions”), external control (items 11–21, including “I could influence the procedures that were carried out”), and support (items 22–33, including “The staff encouraged me to try new ways of coping”). Each item is rated on a 5-point Likert scale, from “strongly disagree” to “strongly agree”. The maximum score is 165, and higher scores on each subscale indicate greater levels of support and control. It is important to note that items 1, 2, 6, 7, 10, 14, 17, 28, 29, and 33 are reverse-scored when interpreting the results [36].

For distribution of the questionnaire, various associations related to birth and the postpartum period, as well as breastfeeding and parenting support groups, were contacted throughout Spain. Collaborating healthcare staff, including midwives, nurses, and doctors, distributed the questionnaire in several areas of the hospital, including maternity wards, postpartum wards, and the delivery room, as well as during postpartum and well-child check-ups at health centres, where mothers bring their newborns for follow-up appointments. The questionnaire was also distributed through various midwifery associations in Spain so they could distribute it to women through their members. The recruitment period was set between May and October 2025. Once participants were selected and agreed to participate, they were given instructions on how to complete the questionnaire. They had the support of the healthcare professional at their respective clinic or service to resolve any doubts or questions that might arise during the process. In addition, participating professionals had access to a WhatsApp group to ask questions and ensure a consistent response across all participating centres. The sampling method was non-probabilistic.

### 2.3. Statistical Analysis

Absolute and relative frequencies were used to describe qualitative variables, and the mean and standard deviation (SD) were used to describe quantitative sociodemographic data. To determine the scale’s validity, content validity, construct validity, and construct-related validity evidence were assessed. Regarding content validity, the relevance of the items was analysed by determining the Content Validity Index (CVI) [37], where values greater than 0.80 should be obtained, as Davis suggested [38].

An Exploratory Factor Analysis (EFA) was chosen to assess construct validity by determining the underlying factors through principal component analysis (PCA). PCA aims to simplify datasets to principal components that retain as much of the original information as possible. This is achieved by transforming potentially correlated variables into a reduced set of new variables, called principal components. Before performing the EFA, we analysed the Kaiser–Meyer–Olkin (KMO) test and Bartlett’s test of sphericity to determine if this analysis was appropriate. For it to be appropriate, the KMO value had to be above 0.6 and as close to 1 as possible, and Bartlett’s test of sphericity, a statistical hypothesis test, had to have a *p*-value less than 0.05 to reject the null hypothesis of sphericity and ensure that the factor model was adequate to explain the data. In the EFA, Varimax rotation was used, and three extraction factors were established based on the original scale. To determine the number of factors to retain, the Kaiser criterion was applied, which consists of retaining those factors whose eigenvalue is greater than one [39].

Construct-related validity evidence was examined by testing theoretically expected associations between SCIB scores (support and control during birth) and obstetric/neonatal variables (e.g., labour induction, mode of delivery, foetal complications, neonatal admission, and maternal involvement in the birthing process). Group comparisons and bivariate analyses were performed using Pearson’s chi-squared test or Fisher’s exact test for qualitative variables, and Student’s *t*-test for quantitative variables, as appropriate. Statistical significance was set at *p* < 0.05.

In addition, the relationship with the BSS-R (Birth Satisfaction Scale–Revised) and the GAD-7 (Generalised Anxiety Disorder Scale) was also studied. Both satisfaction with the birthing process and perceived anxiety levels can be closely related to the degree of control and support during labour. Accordingly, correlations with the BSS-R were interpreted as convergent validity evidence, whereas correlations with the GAD-7 were interpreted as discriminant/divergent validity evidence.

Reliability analysis was performed using Cronbach’s alpha (α) to assess internal consistency (IC). The IC indicates the extent to which the questionnaire items are correlated, how well they fit together, and how well they measure the same concept. α is one of the most widely used measures to assess the reliability of a scale [39,40]. Its values range from 0 to 1. The commonly accepted rule is to consider α > 0.9 as excellent, α > 0.8 as good, α > 0.7 as acceptable, α > 0.6 as questionable, α > 0.5 as poor, and α < 0.5 as unacceptable.

The IBM SPSS Amos statistical software was used to perform confirmatory factor analysis and evaluate the model’s fit. Various fit measures were employed: for absolute fit, the Chi-square test and root mean square error of approximation (RMSEA) were analysed; for incremental fit, the comparative fit index (CFI), Tucker–Lewis index (TLI), and normalised fit index (NFI) were used; and for parsimony fit measures, the parsimony ratio (PRATIO), comparative parsimony fit index (PCFI), and Akaike information criterion (AIC) were employed. The interpretation of these indices was carried out based on the critical values recommended in the literature, which indicate that values greater than 0.90 are acceptable for TLI, CFI, and PRATIO; greater than 0.80 for PCFI and PNFI; and values below 0.08 for RMSEA and the minimum possible value for AIC [41].

Finally, temporal reliability was studied using a test–retest reliability test. To assess this property, the intraclass correlation coefficient (ICC) was used. This was calculated using a two-factor mixed-effects model, which analysed both agreement and absolute agreement. In this case, the questionnaire was re-administered after 72 h to a randomly selected subgroup of participating women to complete this analysis, using the same administration format and instructions; participants were not provided with their previous responses. According to Fleiss’s criteria, ICC values above 0.9 are considered excellent [42].

## 3. Results

### 3.1. Characteristics of Participants

A total of 302 women participated, with a mean age of 35.2 years (SD = 4.18), of whom 65.9% (199) were married; 52% (157) of the women were primiparous, and 11.3% (34) used assisted reproductive technologies.

Regarding prenatal care, 43.7% (132) received care in the public sector. As for childbirth, the birth plan was followed in 35.4% (107) of cases; 46.0% (139) of participants had spontaneous births. The majority, 79.8% (241), did not experience postpartum complications, and 26.5% (80) expressed being quite satisfied with the entire process. The remaining sociodemographic data are shown in Table 1.

### 3.2. SCIB Scale Results

The mean overall SCIB scale score was 113.86 (SD = 27.91). The mean score for internal locus of control was 34.18 (SD = 8.68), while the mean score for external locus of control was 35.31 (SD = 11.82). The mean score for the support dimension was 44.37 (SD = 12.27).

### 3.3. Content Validity

To ensure the relevance and cultural appropriateness of the translated version of the Support and Control in Birth Scale (SCIB), its content validity was assessed using a Delphi panel of experts. Considering a minimum of 0.80 as the consensus criterion for item acceptance, and prior to review, modification, or elimination of items, a Content Validity Index (CVI) < 0.80 was obtained for the adapted 33-item version. This prompted the exploration of a reduced version.

### 3.4. Psychometric Properties of the SCIB Scale

#### 3.4.1. Factor Construct Validity

The KMO test yielded a value of 0.925, and Bartlett’s test of sphericity was *p* < 0.001; therefore, an exploratory factor analysis (EFA) was performed. The first component, comprising items 1 to 10, explained 15.61% of the rotated variance and corresponds to an internal locus of control. The second component, grouping items 11 to 21, explained 19.28% of the rotated variance and represents an external locus of control. Finally, the third component, consisting of items 22 to 33, explained 21.60% of the rotated variance and is related to support. The three principal components explained 56.49% of the cumulative variance. The rotated component matrix is shown in Table 2. Furthermore, all anti-image diagonal correlations showed values greater than 0.737.

#### 3.4.2. Criterion Validity

For the external criterion validity analysis, the relationships among variables that could influence maternal support and control, and their association with the SCIB scale, were examined. As shown in Table 3, statistically significant associations (*p* ≤ 0.05) were found with several variables. Among these, variables related to pregnancy and birth information showed a statistically significant association with whether the last pregnancy was planned (*p* = 0.006), whether it was considered high-risk (*p* < 0.001), how the last labour began (*p* < 0.001), the type of birth (*p* < 0.001), whether the birth plan was followed (*p* < 0.001), whether an epidural was required (*p* = 0.003), and the mother’s active participation in the process (*p* < 0.001). Regarding postpartum complications and outcomes, the presence of postpartum complications (*p* < 0.001) and hospital admission of the baby (*p* = 0.003) were found to be statistically significant.

On the other hand, the variables related to associated factors and risk conditions included aspects such as the presence of foetal diseases during pregnancy (*p* = 0.005), as well as the degree of satisfaction with the prenatal care during the last pregnancy (*p* < 0.001), and overall satisfaction with the pregnancy process (*p* < 0.001). Other variables that may influence the maternal experience and that correspond to related aspects, such as the presence of stressful events and whether the woman received professional mental health support, did not show a statistically significant relationship. Finally, the convergent validity between the SCIB scale and the BSS-R and GAD-7 scales was analysed using Pearson’s correlation coefficient, which showed a statistically significant relationship (*p* < 0.001; see Table 4).

#### 3.4.3. Internal Consistency

To assess internal consistency, the alpha coefficient (α) of the total questionnaire was used, as well as that of each of the dimensions identified through the EFA. For the total scale, Cronbach’s alpha was 0.95. The alpha coefficients for the “support,” “external control,” and “internal control” subscales were 0.88, 0.92, and 0.93, respectively, as shown in Table 2.

#### 3.4.4. Temporal Stability

The reliability of the measurements was assessed using the intraclass correlation coefficient (ICC) with a two-way mixed-effects model. The analysis was performed using two definitions: consistency and absolute agreement. For consistency, the results showed excellent reliability, with an ICC for individual measures of 0.995 (95% CI: 0.990–0.998; F (30,30) = 399,106; *p* < 0.001) and for average measures of 0.997 (95% CI: 0.995–0.999; F (30,30) = 399,106; *p* < 0.001). Under the definition of absolute agreement, the values were identical (ICC = 0.995 for individual measurements, 95% CI: 0.990–0.998, and ICC = 0.997 for average measurements, 95% CI: 0.995–0.999; F (30,30) = 399,106; *p* < 0.001), also confirming excellent inter-measurement reliability. In both cases, the ICC values exceed the threshold of 0.90, which, according to Koo and Li (2016) [42], indicates excellent consistency and agreement among the observations made.

#### 3.4.5. Confirmatory Factor Analysis

**Figure 1:**
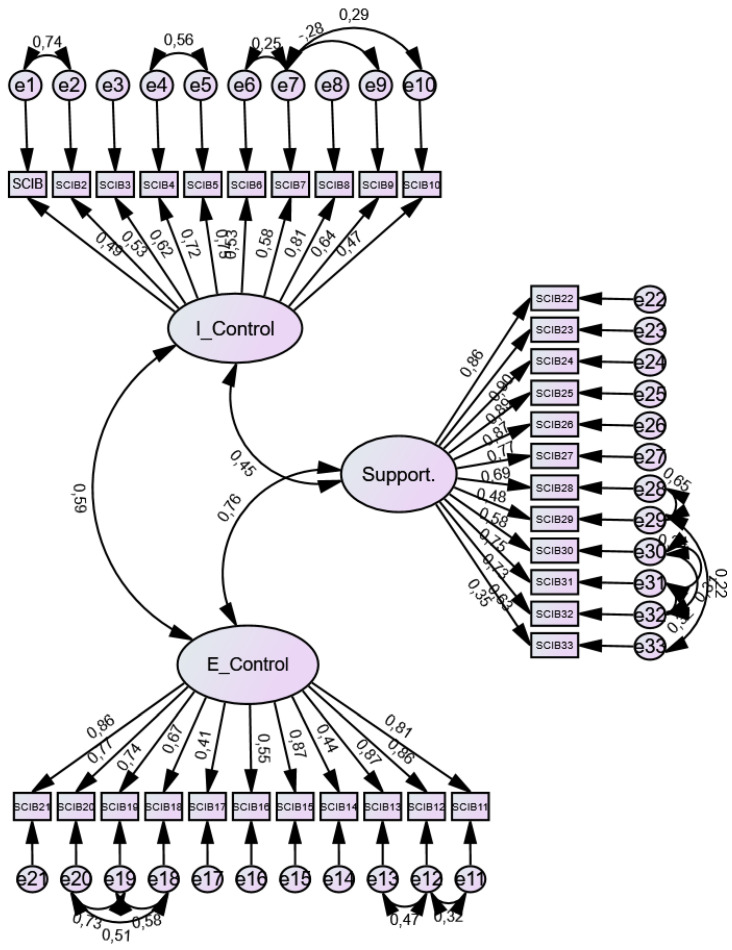
Path Diagram SCIB.

After performing confirmatory factor analysis, a good model fit was observed with the absolute fit index: Chi-square < 0.05, Chi-square/DF (2.36), RMSEA (0.07); the incremental fit indices: TLI (0.91), CFI (0.92), NFI (0.86); and the parsimonious fit indices PRATIO (0.90) and PCFI (0.83). Table 5 shows all the values for each indicator and the criteria required to confirm the model fit. The path diagram is shown in Figure 1. To improve model fit, correlated residuals were incorporated only when modification indices indicated substantial localized strain, and there was a theoretically plausible explanation (e.g., overlapping wording or very similar item content reflecting shared method variance). Importantly, residual correlations were restricted within the same latent factor to avoid compromising the conceptual distinctiveness of the three dimensions. These modifications are interpreted as accounting for shared phrasing/content and should be further examined in independent Spanish samples to assess their robustness and implications for cross-cultural comparability.

#### 3.4.6. Reduced Version SCIB—24 Items

During the content validity assessment process using the Delphi method, participating experts were asked not only to evaluate the relevance and clarity of each item but also to identify, among the 33 items, those that presented a high degree of conceptual or semantic overlap. This procedure aimed to optimise the instrument by reducing content redundancy and facilitating the development of a shorter version of the scale. This, in turn, sought to increase its clinical applicability, facilitating its use in healthcare settings without compromising the representativeness of the constructs being assessed. Following the experts’ evaluations, a high degree of agreement was reached regarding the suitability of the items. Only items 10, 11, 13, 18, 20, 27, 29, and 33 were excluded due to their low scores, resulting in a first version of the questionnaire with 24 items (Supplementary Tables S2 and S3). Item reduction followed a two-step decision process. First, the Delphi panel prioritized content criteria (relevance, clarity, and detection of semantic overlap/redundancy) to preserve construct coverage while improving feasibility. Second, we verified empirically that retained items showed adequate factor loadings and that the resulting 24-item version preserved the original three-factor structure in EFA and achieved acceptable fit in CFA. Finally, the very high correlation between the 33-item and 24-item versions (r > 0.90) supports score comparability.

**Figure 2:**
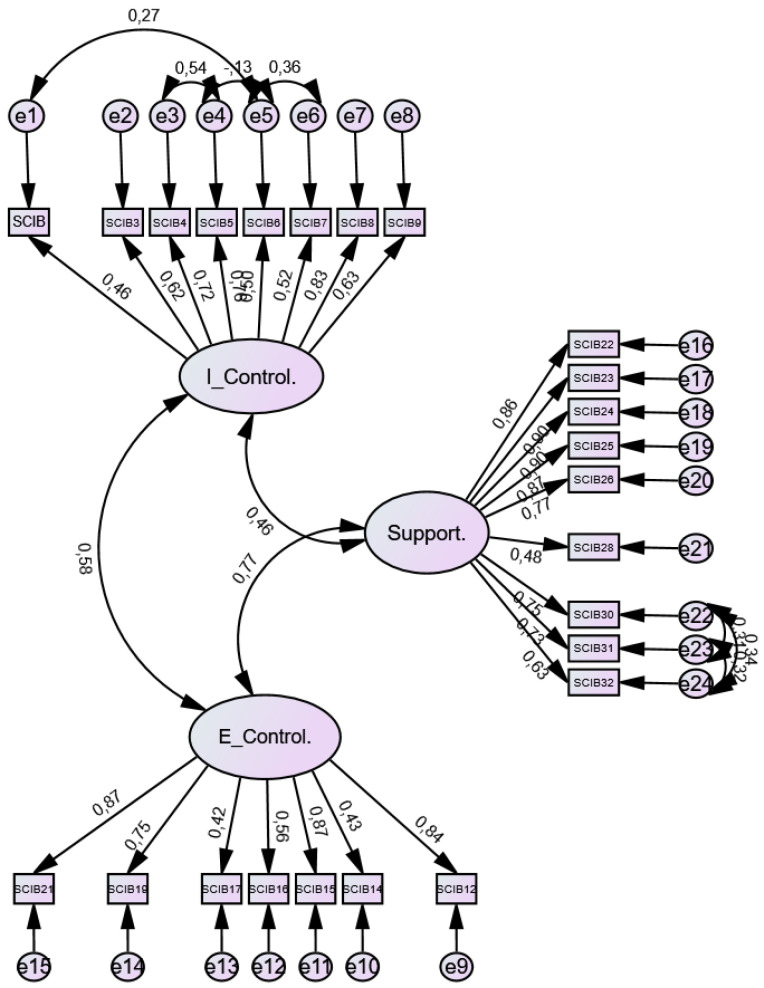
Path Diagram SCIB—Short version 24 items.

This questionnaire was evaluated a second time by experts, who approved the suitability of all items. An IVC value ≥ 0.80 was obtained for the reduced version. Furthermore, the 24-item short version of the instrument proved more efficient in terms of parsimony and psychometric performance, maintaining a clear three-factor structure and appropriate factor loadings. The first component, comprising items 1–8, explained 11.60% of the rotated variance and corresponds to the internal locus of control. The second component, grouping items 9–15, explained 16.79% of the rotated variance and represents the external locus of control. Finally, the third component, consisting of items 16–24, explained 30.97% of the rotated variance and is related to support. The three main components explained 59.36% of the cumulative variance. To assess internal consistency, the alpha coefficient (α) of the total questionnaire was used, as well as that of each of the dimensions identified through the exploratory factor analysis (EFA). For the total scale, Cronbach’s alpha was 0.935. The alpha coefficients for the “support,” “external control,” and “internal control” subscales were 0.85, 0.86, and 0.93, respectively. Furthermore, the degree of correlation between the 33-item and 24-item versions was greater than 0.9, both overall and across the three subscales. After performing the confirmatory factor analysis, a good fit of the model was observed in the absolute fit index: Chi-square < 0.05, Chi-square/DF (2.46), RMSEA (0.07). The incremental fit indices are: TLI (0.92), CFI (0.92), NFI (0.88), and the parsimonious fit index PRATIO (0.88) and PCFI (0.81). Table 6 shows all the values for each indicator and the criteria required to confirm model fit. The path diagram is shown in Figure 2.

## 4. Discussion

The SCIB scale, with expert evaluation and adequate psychometric properties, assesses maternal perceptions of support and control during birth. It demonstrates content, construct, and criterion validity, convergence, good model fit, reliability, and temporal stability, making it a valid and reliable tool for use with postpartum women in Spain.

Regarding potential limitations, there could be selection bias due to non-responses, although there is no evidence to suggest that this bias affected the results. Furthermore, the questionnaire was administered anonymously, which encouraged honest responses from participants. Although non-probability sampling was used, recruitment primarily occurred in hospitals and health centers during routine postpartum follow-ups. This clinical approach ensured a diverse sample and helped mitigate self-selection bias. Additionally, while a six-month postpartum inclusion period may introduce recall bias as perceptions evolve over time, the scale’s high temporal stability suggests that it remains a reliable tool within this timeframe. However, the scale’s high temporal stability suggests it remains a reliable tool throughout this timeframe. It is important to note that the SCIB is not a trauma-specific instrument, and it is not intended to screen or diagnose postpartum PTSD. Rather, it assesses experiential dimensions (support and control) that may act as indirect risk markers given their documented links with later postpartum psychological adjustment. In the present validation, no PTSD-specific measure was included; therefore, any trauma-related implications should be interpreted cautiously, and future studies should incorporate validated postpartum PTSD symptom measures to test these pathways directly. Moreover, while the observed ICC values indicate excellent short-term stability, the magnitude of these coefficients may be partially influenced by the short retest interval (i.e., potential memory effects and contextual stability). Future studies should examine stability across longer intervals (e.g., 1–2 weeks).

Among its strengths, the sample size (*n* = 302) exceeds commonly used thresholds for factor analysis (participant-to-item ratios) and was supported by sampling adequacy indicators (high KMO and significant Bartlett’s test). However, we acknowledge that for a national validation study, this sample remains moderate, and larger multi-site samples would further strengthen the precision of estimates, the robustness of the factorial structure, and subgroup analyses. In addition, given the tool’s adequate psychometric properties, this study proposes a reduced 24-item version that maintains the original scale’s 3-component structure and exhibits excellent internal consistency, facilitating its application in clinical practice.

In our study, the sample used for validation was similar to that of the Turkish version [28], is smaller than that of the Persian [29] and Italian [30], but larger than the Chinese version [11]. The variance of the Spanish version (56.49%) falls within a similar range to the original [27] and the Persian version [29] (55% and 63.1%, respectively), indicating that in these cultural contexts the scale maintains its ability to capture related perceptions. On the other hand, the Turkish [28] (42.85%) exhibits significantly lower variance, suggesting that certain aspects of the construct are less relevant or are interpreted differently in that culture. The absence of exploratory factor analysis in the Chinese [11] version limits comparability with this version.

The SCIB scale differs from other traditional tools for assessing control during labour, which are unidimensional and focused on specific aspects. For example, the W-DEQ scale, created by Wijma et al. [24], focuses on the assessment of fear during and after birth; the Parental Self-Efficacy (PSE) scale, by Brand et al. [43], addresses related aspects such as the perception of maternal autonomy. Likewise, Dencker’s Childbirth Experience Questionnaire (CEQ) scale [26], designed to assess the birth experience more holistically, across various dimensions such as self-capacity, professional support, perceived safety, and participation, the scale was initially applied only to first-time mothers and was revised in 2020, the CEQ-2 [44], removing pain-related items due to model fit issues.

In contrast, the SCIB scale allows for a measurement that encompasses multiple dimensions across three main areas. First, internal control, which includes aspects such as emotions, thoughts, behaviour, pain, and physical functioning. Second, external control, which includes elements such as pain relief, information received, the environment in which the birth takes place, the decisions and procedures performed, and the outcome of the birth itself. Finally, support received, which includes guidance and coping during labour, the attitude of healthcare staff, empathy, encouragement, active listening, informational support, and assistance with pain relief [27]. The literature indicates that the perception of control during birth is multifactorial and plays an important role in the overall birth experience [45,46,47,48]. This reinforces the need for the tool to be multidimensional. The relevance of capturing these subjective perceptions is highlighted by recent research, such as the study by Di Gesto et al. [49], which demonstrates that birth-related experiences are significant predictors of later psychological adjustment, including postpartum PTSD symptoms and breastfeeding intentions. Consequently, the SCIB dimensions act as indirect markers of psychopathological risk, where negative perceptions serve as precursors to adverse psychological morbidity.

In the present study, various aspects of pregnancy and childbirth, such as whether the pregnancy was planned, considered high-risk, its onset, type, adherence to the planned delivery, the need for an epidural, and the mother’s active participation, showed a relationship with the perception of control and support. Likewise, postpartum complications, foetal illnesses, hospital admission, and satisfaction with the prenatal care and the pregnancy process influence the overall perception of the maternal experience. In line with these findings, Volkert et al. [7], in their study involving a sample of 1102 women, demonstrated that foetal and neonatal complications can decrease maternal satisfaction and negatively affect the birth experience. However, a systematic review on the subjective perception of the birth experience. Chabbert et al. [50] found that other obstetric factors, such as epidural analgesia and the type of birth, were not a conclusive influence on maternal satisfaction. These results do not coincide with ours, since in our study, the type of birth and the need for epidural analgesia were indeed related to maternal satisfaction.

Our results also indicate that women’s participation in decision-making is fundamental; this aligns with the recommendations of the World Health Organization, which emphasise the importance of adequate education and staff support for a positive birth experience [51]. Adherence to and monitoring of the expectations established in the birth plan are important factors for a positive birth experience. As Christiaens & Bracke [52] and Larkin et al. [18] note, adhering to the birth plan increases the perception of control and support during the process, which can reduce the caesarean section rate [53] and better neonatal outcomes [53] compared to women whose birth plan is not followed.

The Spanish version of the SCIB scale demonstrated excellent internal consistency (Cronbach’s α = 0.95), an identical value to that reported for the original scale by Ford et al. [27] and in the Persian adaptation [29]. This coefficient places the Spanish adaptation above other cultural adaptations [11,28,30], which reported values lower than α= 0.90. Regarding the specific dimensions, the original scale [27] obtained higher α values in each dimension, standing out from the other versions. However, in the internal control dimension, the Spanish version outperformed the original (α = 0.93 vs. α = 0.86). The temporal stability of the Spanish version (ICC = 0.995) surpasses that reported in other identified instruments [11,28,29,30]; however, it was not possible to establish a comparison with the original version [27], since its temporal stability was not evaluated.

While the factorial validation of the scale confirmed the three-factor structure postulated in the initial 33-item version (as observed in the EFA), it revealed an opportunity to optimise the instrument for efficiency and parsimony. Subsequent psychometric analysis demonstrated that the 24-item short version maintains the three-component structure of the original scale (internal locus of control, external locus of control, and support), exhibits excellent internal consistency (α = 0.935), and shows a high degree of correlation with the 33-item version. Furthermore, this abbreviated version of the scale is considerably more practical, as it reduces administration time and facilitates its integration into clinical settings without sacrificing measurement quality.

The validation of the Support and Control in Birth Scale (SCIB) in Spanish women demonstrated adequate psychometric properties. Adaptation to the Spanish context makes the SCIB a valid and reliable instrument for assessing women’s perceptions of the support received and the degree of control experienced during childbirth. Furthermore, the use of the abbreviated version is recommended, as it maintains the psychometric robustness of the original instrument and offers a more agile, practical, and efficient tool for application in clinical and research settings.

Beyond its technical performance, the scale has clinical implications; it enables the early screening of women perceiving low levels of support or control, allowing for timely interventions to improve maternal psychological outcomes. Moreover, its systematic implementation serves as a necessary metric for evaluating the quality of maternity care and informing the development of evidence-based health policies that promote a birthing process respecting maternal rights and international standards.

## 5. Conclusions

The validation of the Support and Control in Birth Scale (SCIB) in Spanish women demonstrated adequate psychometric properties, and the short version is recommended for use.

## Figures and Tables

**Table 1:** Characteristics of the sample included in a validation study of the Spanish questionnaire for the SCIB scale.

Variable	Total	Variable	Total
	(*n*) %		(*n*) %
**Maternal age**		**Number of pregnancy visits**	
Mean (SD)	35.2 (4.18)	<7	95 (31.5)
**SCIB Scale**		7–10	138 (45.7)
Mean (SD)	113.86 (27.91)	>10	69 (22.8)
Internal control	34.18 (8.68)	**Satisfaction degree with pregnancy follow-up**	
External control	35.31 (11.82)	Not at all	5 (1.7)
Support	44.37 (12.27)	Low satisfaction	31 (10.3)
**Marital Status**		Satisfied	85 (28.1)
Single	53 (17.5)	Quite satisfied	77 (25.5)
Married	199 (65.9)	Highly satisfied	104 (34.4)
Unmarried partner	46 (15.2)	**Worked during last pregnancy**	
Divorced	3 (1.0)	No	50 (16.6)
Widowed	1 (0.3)	Yes	252 (83.4)
**Nationality**		**Health issues during last pregnancy**	
Spanish	292 (96.7)	No	183 (60.6)
Other	10 (3.3)	Yes	119 (39.4)
**Belongs to a religion**		**Setting where pregnancy was followed up**	
No	148 (49.0)	Public	132 (43.7)
Yes, but not practising	104 (34.4)	Private	17 (5.6)
Yes, and practising	50 (16.6)	Mixed (Public mainly)	94 (31.1)
**Current illness**		Mixed (Private mainly)	28 (9.3)
No	241 (79.8)	Mixed (Equally)	31 (10.3)
Yes	61 (20.2)	**Birth plan**	
**Type of birth**		No	157 (52.0)
Vaginal	181 (59.9)	Yes, respected	107 (35.4)
Instrumental	55 (18.2)	Yes, but not respected	38 (12.6)
Elective C/S	15 (5.0)	**Epidural**	
Emergency C/S	51 (16.9)	No, maternal choice	31 (10.3)
**Current medical treatment**		No, medical reason	4 (1.3)
No	242 (80.1)	No, other reasons	18 (6.0)
Yes	60 (19.9)	Yes	249 (82.5)
**Family history of suicide**		**Non-pharmacological analgesia**	
No	248 (82.1)	No	197 (65.2)
Yes	54 (17.9)	Yes	105 (34.8)
**Stressful event during last year**		**Satisfaction degree with postpartum care**	
No	189 (62.6)	Not at all	7 (2.3)
Yes	113 (37.4)	Low satisfaction	28 (9.3)
**Any previous mental health treatment**		Satisfied	54 (17.9)
No	125 (41.4)	Quite satisfied	80 (26.5)
Yes	177 (58.6)	Highly satisfied	133 (44.0)
**Admission to a mental health unit**		**Postpartum complications**	
No	298 (98.7)	No	241 (79.8)
Yes	4 (1.3)	Yes	53 (17.5)
**Number of pregnancies (including current)**		Unsure	8 (2.6)
1	157 (52.0)	**Planned pregnancy**	
2	90 (29.8)	No	29 (9.6)
3	37 (12.3)	Yes	273 (90.4)
4	8 (2.6)	**Pregnancy Risk**	
5	7 (2.3)	Low risk	227 (75.2)
6	1 (0.3)	High risk	75 (24.8)
7	2 (0.7)	**Perineal tear**	
**Active participation during birth**		No	122 (40.4)
No, by choice	9 (3.0)	Episiotomy	49 (16.2)
No, not possible	67 (22.2)	Spontaneous tear (any degree)	130 (43.0)
Yes	226 (74.8)	Episiotomy + tear	1 (0.3)

**Table 2:** Rotated component matrix.

Items	Support	External Control	Internal Control
1. The pain was too great for me to gain control over it.			0.704
2. I was overcome by the pain.			0.757
3. I was able to control my reactions to the pain.			0.612
4. I was mentally calm.			0.647
5. I was in control of my emotions.			0.663
6. I felt my body was on a mission that I could not control.			0.661
7. Negative feelings overwhelmed me.			0.599
8. I gained control by working with my body.			0.694
9. I could control the sounds I was making.			0.577
10. I behaved in a way not like myself.			0.604
11. I had control over when procedures happened.		0.658	
12. I could influence which procedures were carried out.		0.762	
13. I decided whether procedures were carried out or not.		0.788	
14. The people in the room took control.		0.495	
15. I had control over the decisions that were made.		0.799	
16. I could get up and move around as much as I wanted.		0.461	
17. People coming in and out of the room were beyond my control.		0.316	
18. I chose whether I was given information or not.		0.634	
19. I could decide when I received information.		0.724	
20. I had control over what information I was given.		0.727	
21. I felt I had control over the way my baby was finally born.		0.700	
22. The staff helped me find the energy to continue when I wanted to give up.	0.782		
23. The staff seemed to know instinctively what I wanted or needed.	0.804		
24. The staff went out of their way to try to keep me comfortable.	0.832		
25. The staff encouraged me to try new ways of coping.	0.838		
26. The staff realised the pain I was in.	0.809		
27. The staff encouraged me not to fight against what my body was doing.	0.733		
28. I felt the staff had their own agenda.	0.499		
29. I felt like the staff tried to move things along for their own convenience.	0.569		
30. I was given time to ask questions.	0.659		
31. The staff helped me to try different positions.	0.603		
32. The staff stopped doing something if I asked them to stop.	0.530		
33. The staff dismissed things I said to them.	0.378		
**Eigenvalues**	7.12	6.36	5.15
**Rotated variance (%)**	21.60	19.28	15.61
**Unrotated variance (%)**	40.62	10.35	5.51
**Cumulative variance (%)**	56.49		
**Cronbach’s alpha**	0.88	0.92	0.93
**Cronbach’s alpha SCIB**	0.951		

**Table 3:** Relationship between different variables and the maternal control and support scale.

Items	Internal Control		External Control		Support		SCIB	
	Mean (SD)	*p* Value	Mean (SD)	*p* Value	Mean (SD)	*p* Value	Mean (SD)	*p* Value
**Parity**		0.855		0.040		0.509		0.224
Primiparous	34.12 (8.97)		34.40 (11.90)		44.07 (12.45)		112.59 (28.01)	
Multiparous	34.32 (8.01)		37.46 (11.43)		45.09 (11.89)		116.87 (27.61)	
**Wanted pregnancy**		**0.016**		0.117		**0.002**		**0.006**
No	30.48 (10.25)		32.03 (13.18)		37.79 (15.05)		100.31 (36.12)	
Yes	34.58 (8.42)		35.66 (11.65)		45.07 (11.76)		115.30 (26.57)	
**Stressful event last year**		**0.027**		**0.010**		0.131		0.014
No	35.03 (8.55)		36.66 (11.59)		45.19 (11.46)		116.90 (26.69)	
Yes	32.75 (8.73)		33.04 (11.91)		42.99 (13.45)		108.79 (29.27)	
**Current illness**		0.691		0.952		0.776		0.981
No	34.28 (8.86)		35.33 (11.83)		44.26 (12.17)		113.88 (87.84)	
Yes	33.78 (7.95)		35.23 (11.91)		44.77 (12.73)		113.78 (28.40)	
**Previous mental health treatment**		0.312		0.029		0.466		0.119
No	34.78 (8.68)		37.08 (11.27)		44.98 (11.31)		116.84 (26.30)	
Yes	33.76 (8.68)		34.06 (12.07)		43.93 (12.92)		111.75 (28.88)	
**Pregnancy risk**		**0.002**		**<0.001**		**0.006**		**<0.001**
Low risk	35.06 (8.15)		36.79 (11.33)		45.48 (11.48)		117.32 (25.79)	
High risk	31.53 (9.70)		30.84 (12.25)		41.03 (13.96)		103.40 (31.48)	
**Antenatal classes**		0.966		0.772		0.876		0.859
No	34.14 (8.62)		35.67 (12.69)		44.57 (11.90)		114.39 (28.43)	
Yes	34.19 (8.72)		35.20 (11.58)		44.31 (12.41)		113.71 (27.81)	
**Onset of last labour**		**<0.001**		**<0.001**		**<0.001**		**<0.001**
Spontaneous	36.27 (7.96)		39.73 (10.47)		47.85 (10.27)		123.84 (23.67)	
Induced	32.70 (8.86)		32.84 (11.54)		42.58 (13.03)		108.11 (28.56)	
Elective C/S	33.17 (9.34)		24.58 (8.55)		33.00 (10.49)		90.75 (28.89)	
Urgent C/S	27.40 (8.19)		21.70 (9.02)		34.90 (11.53)		84.00 (22.11)	
**Type of birth**		**<0.001**		**<0.001**		**<0.001**		**<0.001**
Normal	35.93 (8.14)		40.19 (9.76)		48.54 (9.92)		124.66 (22.83)	
Instrumental	31.33 (9.16)		28.35 (10.84)		38.89 (13.33)		98.56 (27.92)	
Elective C/S	34.00 (8.66)		25.87 (8.72)		34.93 (11.04)		94.80 (22.45)	
Urgent C/S	31.12 (8.61)		28.27 (11.56)		38.27 (12.87)		97.67 (27.84)	
**Birth plan**		**0.004**		**<0.001**		**<0.001**		**<0.001**
No	34.17 (8.75)		35.81 (11.45)		45.08 (11.42)		115.06 (26.74)	
Yes, and respected	35.64 (8.21)		38.71 (9.74)		48.67 (8.04)		123.02 (20.04)	
Yes, but not respected	30.16 (8.60)		23.68 (11.84)		29.32 (14.20)		83.16 (30.94)	
**Active participation in the last birth**		**<0.001**		**<0.001**		**<0.001**		**<0.001**
No, by choice	34.00 (10.09)		33.56 (16.19)		43.78 (13.54)		111.33 (34.42)	
No, not possible	28.63 (8.69)		22.37 (8.45)		33.06 (13.22)		84.06 (23.36)	
Yes	35.84 (7.94)		39.22 (9.57)		47.75 (9.70)		122.80 (22.25)	
**Satisfaction degree with last pregnancy care**		0.031		**0.004**		**<0.001**		**<0.001**
Not at all	28.40 (11.78)		26.00 (17.10)		31.20 (20.68)		85.60 (47.70)	
Little satisfied	32.03 (8.39)		32.48 (12.52)		41.26 (12.71)		105.77 (29.12)	
Satisfied	32.87 (8.76)		32.53 (11.77)		41.26 (13.18)		106.66 (29.06)	
Quite satisfied	36.21 (8.22)		36.91 (10.37)		45.12 (9.57)		118.23 (22.31)	
Very satisfied	34.67 (8.61)		37.69 (11.78)		47.92 (11.61)		120.29 (26.99)	
**Satisfaction degree with last birth care**		**<0.001**		**<0.001**		**<0.001**		**<0.001**
Not at all	27.71 (14.69)		14.29 (2.36)		15.14 (3.18)		57.14 (18.00)	
Little satisfied	25.96 (7.12)		18.64 (6.66)		24.64 (8.79)		69.25 (18.84)	
Satisfied	31.44 (7.74)		31.15 (9.86)		40.28 (9.76)		102.87 (21.10)	
Quite satisfied	33.88 (8.15)		35.11 (8.71)		45.86 (8.15)		114.85 (19.24)	
Very satisfied	37.55 (7.53)		41.74 (9.70)		50.83 (8.43)		130.11 (19.50)	
**Epidural in last birth**		**0.028**		**<0.001**		0.118		**0.003**
No, maternal choice	38.23 (8.22)		42.87 (9.28)		48.65 (10.44)		129.74 (21.78)	
No, medical indication	38.00 (7.79)		44.75 (5.85)		50.75 (7.80)		133.50 (12.01)	
No, other reasons	32.17 (7.02)		32.89 (11.25)		45.56 (9.59)		110.81 (21.78)	
Yes	33.76 (8.74)		34.39 (11.83)		43.65 (12.61)		111.80 (28.48)	
**Issues during last pregnancy**		**0.036**		0.203		0.443		0.126
No	35.03 (8.35)		36.01 (11.45)		44.81 (11.49)		115.85 (26.49)	
Yes	32.88 (9.04)		34.24 (12.36)		43.70 (13.41)		110.82 (29.83)	
**In utero health problem**		0.139		**<0.001**		0.031		**0.005**
No	34.40 (8.70)		35.99 (11.68)		44.83 (11.99)		115.22 (27.53)	
Yes	31.72 (8.18)		27.80 (11.06)		39.32 (14.41)		98.84 (28.33)	
**Postpartum complications**		**0.007**		**0.001**		**0.002**		**<0.001**
No	34.80 (8.54)		36.36 (11.50)		45.37 (11.85)		116.53 (26.44)	
Yes	31.26 (8.84)		30.40 (12.19)		39.68 (13.22)		101.34 (31.35)	
**Neonatal admission to any care unit**		**0.001**		**0.021**		**0.033**		**0.003**
No	34.80 (8.57)		35.92 (11.72)		44.95 (12.05)		115.67 (27.40)	
Yes	30.03 (8.38)		31.23 (11.89)		40.46 (13.18)		101.72 (28.66)	
**Stillbirth**		0.959		0.784		0.212		0.496
No	34.19 (8.70)		35.34 (11.85)		44.50 (12.19)		114.02 (106.17)	
Yes	34.00 (8.63)		34.00 (11.24)		38.17 (15.85)		106.17 (33.69)	

**Bold *p*-value**: statistically significant difference.

**Table 4:** Convergent validity.

Variable	BSS-R	GAD-7	*p*-Value
**Internal** **control**	0.62	−0.32	**<0.001**
**External** **control**	0.73	−0.28	**<0.001**
**Support**	0.73	−0.19	**<0.001**
**SCIB Total**	0.82	−0.30	**<0.001**

**Bold *p*-value:** statistically significant differences. Pearson correlation coefficient.

**Table 5:** Confirmatory Factorial Analysis. Model fit analysis.

Indicators	Reference Criteria	Original Model Estimated Values	Estimated Values After Correlating Errors
**Absolute fit indices**			
Chi-squared	>0.05	**<0.05**	**<0.05**
Chi-Squared/df	<3	4.62	**2.36**
Root mean squared error of approximation (RMSEA)	<0.08	0.11	**0.07**
**Incremental fit indices**			
Tucker–Lewis Index (TLI)	>0.90	0.75	**0.91**
Comparative fit index (CFI)	>0.90	0.77	**0.92**
Normed Fit Index (NFI)	>0.90	0.72	0.86
**Parsimonious fit indices**			
Parsimony ratio (PRATIO)	>0.90	**0.93**	**0.90**
Comparative Fixed Parsimony Index (PCFI)	>0.80	0.71	**0.83**
Parsimony Normed fit Index (PNFI)	>0.80	0.67	0.78
Akaike Information Criterion (AIC)	Minor value	2413.718	1294.281

**Bold**: meets adequate CFA criteria.

**Table 6:** Confirmatory Factorial Analysis. Model fit analysis for SCIB—Short Version—24 items.

Indicators	Reference Criteria	Original Model Estimated Values	Estimated Values After Correlating Errors
**Absolute fit indices**			
Chi-squared	>0.05	**<0.05**	**<0.05**
Chi-squared/df	<3	4.62	**2.40**
Root mean squared error of approximation (RMSEA)	<0.08	0.11	**0.07**
**Incremental fit indices**			
Tucker–Lewis Index (TLI)	>0.90	0.75	**0.92**
Comparative fit index (CFI)	>0.90	0.77	**0.93**
Normed Fit Index (NFI)	>0.90	0.72	0.88
**Parsimonious fit indices**			
Parsimony ratio (PRATIO)	>0.90	**0.93**	0.88
Comparative Fixed Parsimony Index (PCFI)	>0.80	0.71	**0.81**
Parsimony Normed fit Index (PNFI)	>0.80	0.67	0.77
Akaike Information Criterion (AIC)	Minor value	2413.718	697.040

**Bold**: meets adequate CFA criteria.

## Data Availability

The data is available through the correspondence author.

## Supplementary Materials

The following supporting information can be downloaded at: https://www.mdpi.com/article/10.3390/jcm15072495/s1, Table S1: Spanish version of the “Support and Control in Birth” scale (Support and Control in Birth—SCIB); Table S2: Abbreviated SCIB version validated by experts—Short version (24 items); Table S3: Methodological Appendix—Justification for Item Reduction (SCIB—Short Version).
