# Supplementary material for: Adaptation and Validation of the “Support and Control in Birth” (SCIB) Tool in Postpartum Spanish Women
Source: J Clin Med. 2026 Mar 24;15(7):2495. doi: 10.3390/jcm15072495 (PMC13073406; doi:10.3390/jcm15072495)
Supplement: Supplementary file 1 [file jcm-15-02495-s001.zip › jcm-4178866-supplementary.pdf]

Supplementary Table S1. Spanish version of the "Support and Control in Birth" scale (Support and Control in Birth—SCIB).

Versión española 33 ítems de la escala "Apoyo y Control en el Parto" (Support and Control in Birth—SCIB).

A continuación, encontrará declaraciones sobre aspectos relacionados con el apoyo y control en el parto. Con respecto a cada afirmación, marque la opción adecuada para usted entre las siguientes:

1: "Totalmente en desacuerdo"
2: "En desacuerdo"
3: "Ni de acuerdo, ni en desacuerdo"
4: "De acuerdo"
5: "Totalmente de acuerdo".

Código Interno:	1	2	3	4	5
1. El dolor era demasiado fuerte para que pudiera controlarlo					
2. El dolor me superó.					
3. Pude controlar mis reacciones al dolor.					
4. Estaba mentalmente tranquila.					
5. Pude controlar mis emociones.					
6. Sentí que no podía controlar lo que sucedía en mi cuerpo.					
7. Los sentimientos negativos me invadieron.					
8. Las acciones que realizaba con mi cuerpo me ayudaban a mantener el control.					
9. Pude controlar los gritos y ruidos que hacía.					
10. Me comporté de una manera que no parecía yo.					
11. Tenía control sobre el momento en que se realizaban los procedimientos.					
12. Podía influir en qué procedimientos se llevaban a cabo.					
13. Decidía si las intervenciones se realizaban o no.					
14. El personal sanitario tenía el control de la asistencia durante el proceso de parto.					
15. Tenía control sobre las decisiones que se tomaban respecto a mi proceso de parto.					
16. Podía levantarme y moverme tanto como quisiera.					
No controlaba las personas que entraban y salían de la habitación.					
18. Elegí ser informada o no.					
19. Podía decidir qué información recibía y cuándo.					
20. Tenía control sobre qué información me daban o no, y en qué momento.					
21. Sentí que tenía control sobre la forma en que se produjo el nacimiento de mi bebé.					
22. El personal me ayudó y animó a continuar cuando quería rendirme.					
23. El personal parecía saber lo que quería o necesitaba.					
24. El personal hizo todo lo posible para mantenerme cómoda.					
25. El personal me animó a probar y usar nuevas formas para afrontar el proceso de parto (por ejemplo, técnicas de respiración).					
26. El personal era consciente del dolor que sentía.					
27. El personal me animó a no resistirme a lo que estaba sucediendo en mi cuerpo.					
28. Sentí que el personal tenía su propia agenda.					
29. Sentí que el personal actuaba según sus propios intereses.					
30. Me dieron tiempo para hacer preguntas.					
31. El personal me ayudó a probar diferentes posiciones.					
32. El personal dejó de hacer algo si le pedí que parara.					
33. El personal no llevó a cabo o interrumpió algo cuando se lo pedí.					

Supplementary Table S2. Spanish version of the "Support and Control in Birth" scale (Support and Control in Birth—SCIB)—abbreviated version validated by experts—Short version (24 items).

Versión española 24 ítems de la escala "Apoyo y Control en el Parto" (Support and Control in Birth—SCIB).

Instrucciones

A continuación, encontrará afirmaciones sobre apoyo y control durante el parto. Indique su grado de acuerdo marcando una opción de 1 a 5:

1: Totalmente en desacuerdo
2: En desacuerdo
3: Ni de acuerdo ni en desacuerdo
4: De acuerdo
5: Totalmente de acuerdo

Nota: Algunos ítems se puntúan de forma invertida (ver más abajo).

Código Interno:		1	2	3	4	5
**Dimensión A—Control interno (8 ítems)**						
1.	1) El dolor era demasiado fuerte para que pudiera controlarlo. [Invertir]					
2.	3) Pude controlar mis reacciones al dolor.					
3.	4) Estaba mentalmente tranquila.					
4.	5) Pude controlar mis emociones.					
5.	6) Sentí que no podía controlar lo que sucedía en mi cuerpo. [Invertir]					
6.	7) Los sentimientos negativos me invadieron. [Invertir]					
7.	8) Las acciones que realizaba con mi cuerpo me ayudaban a mantener el control.					
8.	9) Pude controlar los gritos y ruidos que hacía.					
**Dimensión B—Control externo (7 ítems)**						
9.	12) Podía influir en qué procedimientos se llevaban a cabo.					
10.	14) El personal sanitario tenía el control de la asistencia durante el proceso de parto. [Invertir]					
11.	15) Tenía control sobre las decisiones que se tomaban respecto a mi proceso de parto.					
12.	16) Podía levantarme y moverme tanto como quisiera.					
13.	17) No controlaba las personas que entraban y salían de la habitación. [Invertir]					
14.	19) Podía decidir qué información recibía y cuándo.					
15.	21) Sentí que tenía control sobre la forma en que se produjo el nacimiento de mi bebé.					
**Dimensión C—Apoyo del personal (9 ítems)**						
16.	22) El personal me ayudó y animó a continuar cuando quería rendirme.					
17.	23) El personal parecía saber lo que quería o necesitaba.					
18.	24) El personal hizo todo lo posible para mantenerme cómoda.					
19.	25) El personal me animó a probar y usar nuevas formas para afrontar el proceso de parto (por ejemplo, técnicas de respiración).					
20.	26) El personal era consciente del dolor que sentía.					
21.	28) Sentí que el personal tenía su propia agenda. [Invertir]					
22.	30) Me dieron tiempo para hacer preguntas.					
23.	31) El personal me ayudó a probar diferentes posiciones.					
24.	32) El personal dejó de hacer algo si le pedí que parara.					

Puntuación

Escala Likert 1–5. Invertir los ítems marcados como [Invertir] usando la transformación: X_invertido = 6 − X.

Para mantener la comparabilidad con la escala original (máximo 165 puntos), utilizar reescalado lineal a 33–165 tanto por dimensión como para el total:

• Dim_reesc_165 = (Suma_dim / (N_dim × 5)) × 165 → rango 33–165

• Total_reesc_165 = (Suma_24 / 120) × 165 → rango 33–165
Donde Suma_dim es la suma (tras invertir) de los ítems de la dimensión, y N_dim es el número de ítems de esa dimensión (A=8, B=7, C=9).

Supplementary Table S3. Methodological Appendix—Justification for Item Reduction (SCIB—Short Version).

The reduction was carried out through content review by a panel of experts in maternal health and research methodology, eliminating semantic overlaps and redundancies while preserving the three-dimensional structure.

Criteria Applied.

- Semantic overlap (items that express the same construct).
- Ambiguity or less clear formulations compared to their retained counterpart.
- Redundancy with items of greater generality/representativeness.
- Balance of content per dimension (internal/external control and support).

Items removed and justification.

Item No.	Summary of writing	Reason for elimination	Consensus Commentary
2	The pain overwhelmed me.	Overlap with 1 (loss of control due to pain).	Option 1 is preferred for clarity.
10	I behaved in a way that didn't seem like myself.	Redundant with 6 (loss of bodily control).	Option 6 best captures the construct.
11	Control over the timing of procedures.	Overlap with 12 (control/influence over procedures).	Option 12 is more balanced.
13	I decided whether interventions were performed.	Redundant/extreme with respect to 12.	Option 12 reflects influence without absolute control.
18	I chose whether or not to be informed.	Overlap with 19 (what and when).	Option 19 integrates content and temporality.
20	Total control over information and timing.	Overlap with 19.	Duplication is avoided; clarity is maintained.
27	I was encouraged not to resist.	Redundant with 22 (emotional support).	Option 22 is retained for generality.
29	I acted according to my interests.	Overlap with 28 (own agenda).	Option 28 is retained for less value bias.
33	Staff did not carry out or interrupt something when I asked them to.	Overlap with 32 (stopping actions).	Option 32 is retained for linguistic naturalness.

Calculation adjustments for comparability (maximum 165 points).

- Reverse items 1, 6, 7, 14, 17, and 28 using the rule X_inv = 6 − X.
- Sum by dimension: A (8 items), B (7 items), C (9 items).
- Rescaling of each dimension: Dim_reesc_165 = (Sum_dim / (N_dim × 5)) × 165 → range 33–165.

- Rescaling of the total (24 items): Total_reesc_165 = (Sum_24 / 120) × 165 → range 33–165.
